# Diuretics and mortality reduction in incident dialysis patients: a two-year observational study

**DOI:** 10.1038/s41598-024-65643-8

**Published:** 2024-11-10

**Authors:** Maxime Ingwiller, Dogan-Firat Bozman, Nans Florens, Damiano Cerasuolo, Cécile Vigneau, Cécile Couchoud, Thierry Hannedouche

**Affiliations:** 1 Department of Nephrology, Hôpitaux Universitaires de Strasbourg, 1 place de l’Hôpital, 67 000 Strasbourg, France; 2 Biostatistics and Clinical Research Unit, CHU Caen, Av. de la Côte de Nacre, 14000 Caen, France; 3 Department of Nephrology, CHU Rennes, 2 rue Henri Le Guilloux, 35000 Rennes, France; 4 Agence de Biomédecine, Registre REIN, 1 avenue du stade de France, 93212 Saint Denis La Plaine Cedex, France; 5 Renal Research Division, AURAL, 4 rue Henri Bergson, 67200 Strasbourg, France

**Keywords:** Chronic dialysis, Hemodialysis, Peritoneal dialysis, Fluid overload, Loop diuretics, Survival, Nephrology, Renal replacement therapy, Haemodialysis

## Abstract

Fluid overload predicts morbidity and mortality in dialysis patients. Diuretics can reduce fluid overload, but their effects on morbi-mortality following inception remain ill-defined. To determine whether diuretics reduce mortality and hospitalization rates in incident dialysis patients in the first 2 years after inception. Adult patients starting dialysis between 2009 and 2015 in the REIN registry were matched with the SNDS database and divided into four subgroups according to loop diuretics exposure: < 5%, 5–50%, 50–80% or > 80% over the observation period of each patient. The incidence of hospitalization was described based on the primary diagnoses of the discharge summaries and codes. In this study, which included 67,736 patients, 53,829 (79.5%) who had varying degrees of diuretic exposure exhibited a significantly lower mortality rate at 2 years compared to those without diuretic exposure (24.8% [95% CI 24.4–25.2], vs 37.5% [95% CI 36.7–38.3] respectively). However, the duration of diuretic exposure significantly impacted outcomes. The all-cause mortality rate at two years was highest in the group with ≥ 80% exposure (68.2% [95% CI 67.3–69.1]), followed by those with 50–80% exposure (15.7% [95% CI 15.0–16.4]), and those with 5–50% exposure (6.6% [95% CI 6.3–7.0]). An inverse probability weighting (IPW) propensity score analysis supported these findings. Stratified analyses showed consistent results regardless of a history of congestive heart failure and were similar for both hemodialysis and peritoneal dialysis patients. Additionally, the number of hospitalizations and length of stay were generally higher in the group with the longest exposure to loop diuretics. Diuretic exposure was generally associated with a lower mortality rate in dialysis patients. However, prolonged exposure (≥ 80%) was linked to an increased risk of mortality and hospitalization. This prolonged exposure may indicate residual diuresis at the cost of chronic fluid overload.

## Introduction

End-stage kidney disease (ESKD) is characterized by sodium and fluid retention, a significant determinant of hypertension, congestive heart failure and cardiovascular mortality^1^.

Among therapeutic measures to prevent or correct sodium overload, using diuretics in ESKD remains controversial. Despite the widespread use of diuretics in non-dialysis CKD patients, these drugs are often discontinued at dialysis initiation. Possible reasons for discontinuation include the misconception that diuretics may no longer be effective, could hasten the decline of residual kidney function (RKF), and that dialysis treatment alone is sufficient for managing fluid overload.

In the hemodialysis CHOICE study, the persistence of RKF reduced the risk of one-year death from all causes, especially cardiovascular deaths^2^. In a small randomized study of 19 patients, Lemes et al. reported that diuretics may have a beneficial effect on the maintenance of RKF and cardiac function through various mechanisms, including maintenance of renal clearance of uremic toxins, attenuation of interdialytic weight gain, reduction of hypotension and myocardial stunning^3^. Van Olden et al. evaluated the effect of maintaining diuretics for one year in hemodialysis patients. They found that high-dose furosemide (500 to 2000 mg/d) increased urine volume (+ 60%), natriuresis and kaliuresis, albeit the response declined over time^4^. In peritoneal dialysis, a small, randomized, controlled trial of 61 incident patients showed that high-dose daily furosemide was associated with higher urine volume and urinary sodium excretion compared with controls at a follow-up of 1 year; however, clinical outcomes were not examined^5^.

The use of loop diuretics was analyzed in 16,420 hemodialysis patients from the international DOPPS cohort^6^. The prevalence of diuretics use was 32.6% at inception but sharply decreased to 10.7% after two years. In prevalent patients, loop diuretics use was lower in the United States (9.2%) compared to Europe (21.5%) and Japan (18.7%). The use of diuretics was associated with lower odds of inter-dialytic weight gain and hyperkalemia, and a two-fold probability of retaining residual diuresis one year after the start of dialysis. Patients on diuretics had a 14% significant reduction in cardiovascular mortality (p ≤ 0.03), but not all-cause mortality (p = 0.12).

In a cohort of 11,297 patients starting hemodialysis in a large US dialysis organization, Sibbel et al. evaluated the impact of continuing versus stopping diuretics on survival, hospitalizations and intradialytic hypotension during the first year of dialysis. They found that continued diuretic therapy significantly reduced the number of hospitalizations and intra-dialytic hypotensions but not deaths at 1 year^7^.

We used the prospective nationwide REIN registry to test the hypothesis that diuretics could reduce morbidity-mortality during the first 2 years after dialysis inception.

## Patients and methods

We linked data from the REIN registry with the National Health Data System (SNDS). The REIN registry collects data on all patients with ESKD in France, including demographic information, initial kidney disease, comorbidities, and dialysis modalities. The SNDS is a medico-administrative database that gathers information on ambulatory care reimbursements, including drug dispensing and hospitalizations. We matched these two anonymized databases using an indirect “deterministic” method based on age, sex, place of residence, place of treatment, date of treatment, and date of death if relevant^8^. This allowed us to identify diuretic treatment and its duration based on the number of boxes dispensed by pharmacies, as well as the frequency, duration, and cause of hospitalizations^9^ and events of interest collected annually in the REIN registry^10^.

Our study included all adult patients (> 18 years) who started dialysis between 2009 and 2015 and could be matched between the two databases. All experiments were performed in accordance with relevant guidelines and regulations. The study was nested within the REIN registry, which has been approved by the Comité Consultatif sur le Traitement de l’Information en matière de Recherche dans le domaine de la Santé (CCTIRS), Commission National Informatique et Liberté (CNIL), and the Scientific Council of the Agence of Biomedicine. All patients gave their written informed consent to participate in the registry.

We extracted initial patient characteristics from the REIN registry, including the number of visits to a nephrologist before starting dialysis, characteristics at initiation such as plasma creatinine, estimated GFR (eGFR) according to MDRD, serum albumin, hemoglobin, and conditions related to initiation such as emergency start, use of a central venous catheter (CVC), or first session in an intensive care unit (ICU). Patients were stratified and analyzed according to their dialysis modality: hemodialysis (HD) or peritoneal dialysis (PD).

After analyzing all diuretic classes, including loop diuretics, thiazides, and potassium-sparing diuretics, our focus shifted exclusively to loop diuretics, representing over 95% of all prescribed diuretics.

Patients were first categorized based on their exposure or non-exposure to loop diuretics. Among those exposed to diuretics, the duration of their prescription over the two years following the start of the study was quantified. These patients were further divided into four subgroups according to their exposure duration: < 5%, 5–50%, 50–80%, or > 80% of the observation period. Patients who either died or received a transplant within the 2 years after starting dialysis were censored at the date of the first event.

The primary endpoint of our study was mortality from all causes and cardiovascular causes. Secondary endpoints included the number of hospitalizations, their motives, and length of stay. We performed a subgroup analysis according to initial dialysis modality (hemodialysis or peritoneal dialysis).

### Statistical analyses

Univariate analyses were performed using the Student test for continuous variables and the Chi2 test for quantitative variables. A value of p < 0.05 was considered statistically significant.

The hospitalization rate was the number of hospital stays relative to the number of people at risk over the period. The total length of hospitalization was the total number of nights spent in the hospital relative to the number of at-risk individuals over the period.

The 95% confidence intervals were calculated from the variance of the rate calculated as follows: number of events/person-at-risk squared. Two rates were considered different if their confidence intervals did not overlap. A one-factor analysis of variance (ANOVA) was used to compare the means in each exposure group with the Fisher F test.

The effect of diuretics on the risk of death was analyzed from a cumulative incidence curve using the Fine and Gray concurrent risk method. The probability of death for a given cause considers that other causes act as competing risks. The death rates were calculated at 12 and 24 months, depending on diuretics exposure. Two death rates were considered different if their confidence intervals did not overlap. A multivariate logistic regression model was performed to assess odds ratio for two years mortality.

We hypothesized that there would be imbalances in patient characteristics among both subdivisions (diuretics vs. no diuretics and diuretics classified into four groups). To address this, we applied a propensity score analysis using the inverse probability weighting (IPW) method. This approach was intended to minimize bias due to measured confounders.

The propensity score for each patient was calculated as a probability from a logistic regression model that included all covariates deemed likely to have affected treatment decisions and mortality—including age, sex, diabetes, BMI, vascular access, congestive heart failure, coronary heart disease, heart rhythm disorders, serum albumin level, start of dialysis with a central venous catheter, dialysis modality (peritoneal dialysis or hemodialysis), cancer, emergency start of dialysis, first dialysis session in an ICU, number of nephrology visits within one year before inception. Stabilized weights were calculated for each patient based on the estimated propensity score. We assessed covariate balance after weighting using the absolute standardized mean difference, whereby the difference above 10% represents a significant imbalance. Cumulative incidence of death curves for the four groups was created with IPW-adjusted cumulative events plots.

The statistics were generated using SAS software and macro %cuminc and using "WeightIt" package in R for the IPW analysis.

## Results

A total of 67,736 adult patients started dialysis between 2009 and 2015. Of those, 18,434 patients failed to meet the criteria for a strong match between REIN and SNDS; 266 were lost to follow-up and were excluded. Therefore, we retained 59,302 patients for analysis. Supplemental Fig. 1 shows the STROBE flowchart diagram.

Among all dialysis patients, 22,186 had no diuretics at dialysis initiation. Of these, 13,907 patients had no exposure to diuretics during the entire two-year follow-up, while 53,829 patients experienced varying degrees of diuretic exposure over time. In this latter group, the prescribed diuretics were predominantly furosemide 500 mg (77.8%) and furosemide 40 mg (16.6%), although individual daily dosages were not available.

Patients exposed to diuretics were divided into four subgroups based on their duration of exposure over the two-year observation period: < 5%, 5–50%, 50–80%, and ≥ 80%. Most patients started their diuretic prescription at or shortly after the inception of the study.

In the entire cohort, 14,810 patients had < 5% exposure (including the 13,907 patients [93.9%] with zero exposure), comprising 13,947 patients on hemodialysis (HD) and 863 on peritoneal dialysis (PD). The 5–50% exposure group included 19,836 patients, with 18,398 on HD and 1438 on PD. The 50–80% exposure group had 10,421 patients, with 9255 on HD and 1166 on PD. The ≥ 80% exposure group consisted of 14,235 patients, including 12,233 on HD and 2002 on PD. A Sankey diagram summarizes the flows of diuretic prescriptions over time and across these groups (Supplemental Fig. 2).

### General patient characteristics

Patients were predominantly male (63.4%), aged 68 years (mean ± 15.4), and overweight (mean BMI 26.4 ± 5.8 kg/m^2^). There were 42.1% of people with diabetes, 25.7% with heart failure, 8.7% in NYHA stages 3–4, and 12% active smokers. The most common causes of initial kidney disease were hypertensive nephropathy (25.8%) and diabetic nephropathy (22.7%). The vast majority of patients maintained walking autonomy (82.6%).

Table 1 illustrates the characteristics of patients based on their exposure to loop diuretics. Overall, patients who were prescribed diuretics had a significantly higher burden of cardiovascular comorbidities compared to those who were not exposed to diuretics.

**Table 1:** Characteristics of patients according to exposure or lack of exposure to diuretics.

Variables	Diuretics (N = 45,395)	No diuretics (N = 13,907)	p
Mean age	68.3 (± 15)	66.9 (± 16.4)	< 0.0001
Men	28,984 (63.8%)	8620 (62%)	< 0.0001
Cause of ESKD			< 0.0001
Hypertensive nephropathy	12,190 (26.8%)	3129 (22%)	
Diabetic nephropathy	11,286 (24.9%)	2181 (15.7%)	
Glomerulonephritis	5244 (11.5%)	1457 (10.5%)	
Polycystic kidney disease	2507 (5.5%)	1048 (7.5%)	
Pyelonephritis	1595 (3.5%)	810 (5.8%)	
Other	5846 (12.9%)	2921 (21%)	
Unknown	6301 (7.9%)	2196 (15.8%)	
Cardiovascular history and other risk factors			
Diabetes	20,354 (45.1%)	4428 (32.1%)	< 0.0001
BMI	26.9 ± 5.9	24.9 ± 5.3	< 0.0001
Stroke/TIA	4777 (10.8%)	1509 (11.2%)	0.2
Coronary heart disease	11,709 (26.7%)	2751 (20.5%)	< 0.0001
Heart rhythm disorders	10,188 (23.1%)	2614 (19.4%)	< 0.0001
Congestive heart failure	11,874 (26.9%)	2917 (21.7%)	< 0.0001
NYHA stages			< 0.0001
Stages 1–2	6961 (16.1%)	1572 (11.9%)	
Stages 3–4	3846 (8.9%)	1054 (8%)	
Peripheral arterial disease	9256 (21.2%)	2184 (16.4%)	< 0.0001
Cancer	4257 (9.6%)	2079 (15.4%)	< 0.0001
Respiratory insufficiency	6642 (15.1%)	1625 (12.1%)	< 0.0001

Table 2 summarizes the characteristics of patients at inception, according to the duration of diuretics exposure. Patients with a < 5% exposure were less overweight than the other groups, with an average BMI of 25.1 kg/m^2^ (p < 0.0001). There was more history of heart failure in the exposure group ≥ 80% (4,252 (30.9%); p < 0.0001), more coronary artery disease (3,904 (28.5%); p < 0.0001), and heart rhythm disorders (3,659 (26.6%); p < 0.0001). The kidney diseases most represented in each group were hypertensive and diabetic nephropathies.

**Table 2:** Initial characteristics of dialysis patients according to the duration of loop diuretic exposure.

	Exposure ≤ 5%	Exposure 5–50%	Exposure 50–80%	Exposure ≥ 80%	p
N (59,302)	14,810	19,836	10,421	14,235	
Men	9151 (61.8%)	12,321 (62.1%)	6733 (64.6%)	9399 (62.0%)	< 0.0001
Mean age	67.0 (± 16.3)	68.0 (± 14.0)	68.7 (± 15.0)	68.3 (± 16.4)	< 0.0001
Cause of ESKD					< 0.0001
Hypertensive nephropathy	3376 (22.8%)	5396 (27.2%)	2847 (27.3%)	3700 (26.0%)	
Diabetic nephropathy	2372 (16.0%)	5428 (27.4%)	2677 (25.7%)	2990 (21.0%)	
Glomerulonephritis	1572 (10.6%)	2158 (10.9%)	1168 (11.2%)	1803 (12.7%)	
Polycystic kidney disease	1100 (7.4%)	949 (4.8%)	567 (5.4%)	939 (6.6%)	
Pyelonephritis	851 (5.7%)	743 (3.7%)	308 (3.0%)	503 (3.5%)	
Other vascular nephropathies	169 (1.1%)	181 (0.9%)	86 (0.8%)	155 (1.1%)	
Other	3059 (20.7%)	2443 (12.3%)	1257 (12.1%)	2008 (14.1%)	
Unknown	2311 (15.6%)	2538 (12.8%)	1511 (14.5%)	2137 (15.0%)	
Cardiovascular risk factors					
Active smoking	1479 (12.2%)	2069 (12.3%)	1081 (12.4%)	1327 (11.2%)	< 0.0001
Diabetes	4795 (32.7%)	9285 (47.0%)	4892 (47.2%)	5810 (41.2%)	< 0.0001
BMI (Kg/m^2^)	25.1 (± 5.4)	27.4 (± 6.2)	26.9 (± 5.8)	26.1 (± 5.4)	< .0001
Stroke/TIA	1599 (11.2%)	2063 (10.7%)	1122 (11.1%)	1502 (10.9%)	< 0.0001
Coronary heart disease	2957 (20.7%)	4831 (25.1%)	2768 (27.4%)	3904 (28.5%)	< 0.0001
Heart rhythm disorders	2781 (19.5%)	3955 (20.5%)	2407 (23.8%)	3659 (26.6%)	< 0.0001
Congestive heart failure	3114 (21.7%)	4696 (24.3%)	2727 (26.9%)	4254 (30.9%)	< 0.0001
NYHA stages					< 0.0001
Stages 1–2	1702 (12.1%)	2994 (15.8%)	1605 (16.2%)	2232 (16.7%)	
Stages 3–4	1103 (7.9%)	1285 (6.8%)	891 (9.0%)	1621 (12.1%)	
Peripheral arterial disease	2118 (15.2%)	3546 (18.8%)	2029 (20.5%)	2714 (20.4%)	< 0.0001
Cancer	2159 (15.2%)	1652 (8.6%)	979 (9.7%)	1546 (11.3%)	
Behavioral disorders	530 (3.9%)	448 (2.4%)	212 (2.2%)	387 (2.9%)	< 0.0001
Walking autonomy					< 0.0001
Total inability	1055 (8.0%)	480 (2.7%)	346 (3.7%)	686 (5.4%)	
Assistance from a 3rd party	1965 (14.9%)	1769 (9.8%)	1142 (12.2%)	1818 (14.4%)	
Total autonomy	10,180 (77.1%)	15,803 (87.5%)	7883 (84.1%)	10,155 (80.2%)	
Respiratory insufficiency	1732 (12.2%)	2785 (14.5%)	1566 (15.6%)	2184 (16.0%)	< 0.0001

Supplemental Tables 1 and 2 shows the characteristics of the patients stratified according to dialysis modality, hemodialysis and peritoneal dialysis, respectively.

### Mortality in dialysis

**Figure 1:**
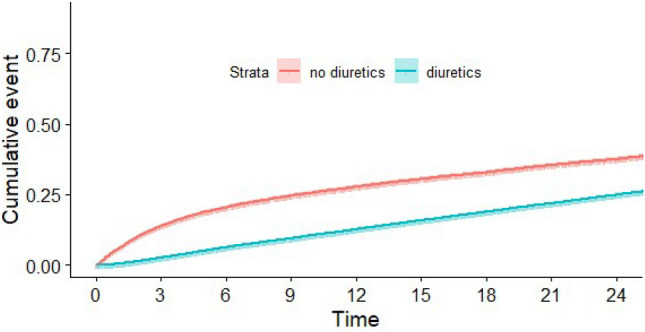
IPW-adjusted cumulative incidence (and 95% confidence interval) of all-cause death, according to loop diuretic exposure or not.

In the entire cohort, mortality at two years was observed in 15,554 incident patients (26.2%). Figure 1 displays the IPW-adjusted cumulative incidence of all-cause deaths up to two years after inception, categorized by exposure to loop diuretics. Despite a higher prevalence of comorbidities, patients receiving loop diuretics had a significantly lower 2-year mortality rate: 24.8% (95% CI 24.4–25.2) compared to 37.5% for patients without diuretic exposure (95% CI 36.7–38.3).

**Figure 2:**
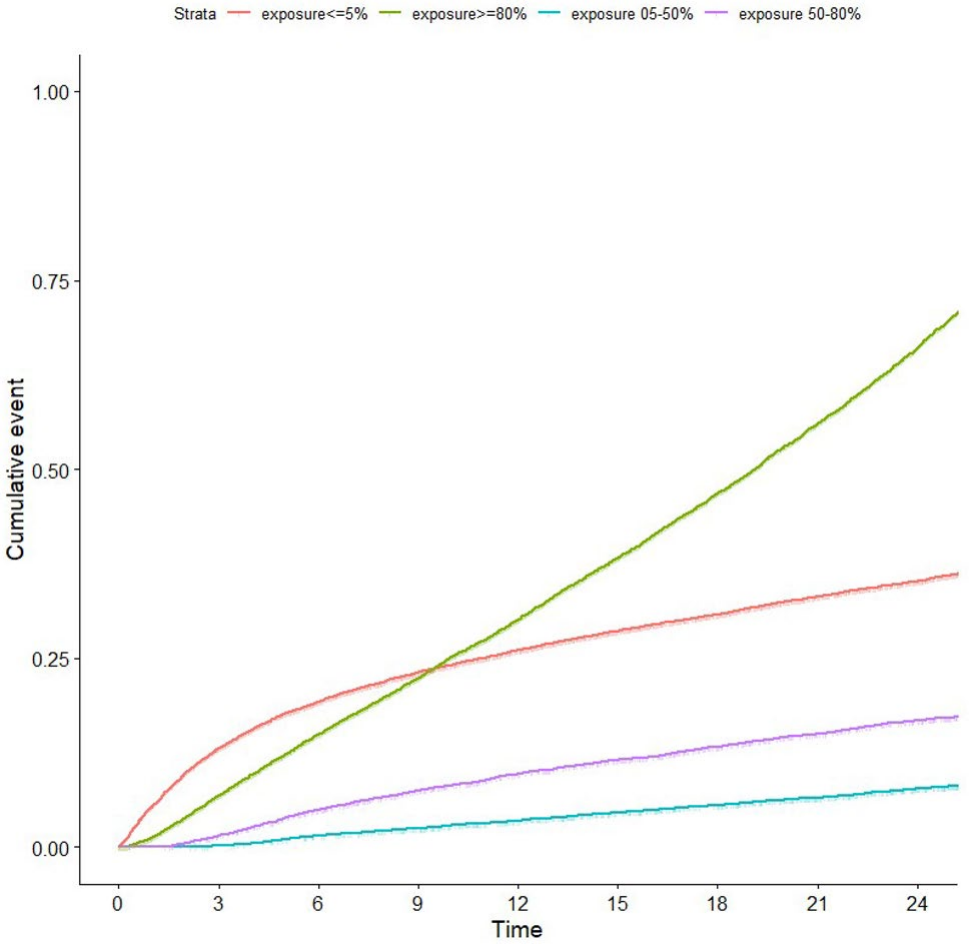
IPW-adjusted cumulative incidence (and 95% confidence interval) of death after inception, according to the duration of loop diuretic exposure.

Figure 2 illustrates the cumulative incidence of all-cause deaths up to two years after the start of dialysis, categorized by the duration of loop diuretic exposure. The one-year mortality rate was highest in the group with the longest exposure (> 80%) at 31.4% (95% CI 30.6–32.2), followed by the < 5% exposure group at 26.0% (95% CI 25.3–26.8), the 50–80% exposure group at 8.8% (95% CI 8.3–9.4), and finally the 5–50% exposure group at 2.8% (95% CI 2.6–3.0).

Similar trends were observed for the two-year mortality rate:

-  > 80% exposure group: 68.2% (95% CI 67.3–69.1),
-  < 5% exposure group: 35.2% (95% CI 34.4–36.0),
- 50–80% exposure group: 15.7% (95% CI 15.0–16.4),
- 5–50% exposure group: 6.6% (95% CI 6.3–7.0).

The adjusted odds ratio for two-year all-cause mortality was also highest in the longest exposure group (≥ 80%): 3.26 (95% CI 2.99–3.54), with the < 5% exposure group serving as the reference category (Table 3).

**Table 3:** Adjusted Odds Ratio for two years all-cause mortality according to the duration of loop diuretic exposure and 95% confidence interval (CI).

Diuretics exposure	Odds ratio	95% CI	p
5–50%*	0.14	[0.12–0.15]	< 2e − 16
50–80%*	0.32	[0.29–0.36]	< 2e − 16
≥ 80%*	3.26	[2.99–3.54]	< 2e − 16
Age	1.06	[1.05–1.06]	< 2e − 16
Sex = F	0.9	[0.83–0.96]	0.00248
Congestive heart failure	1.99	[1.84–2.14]	< 2e − 16
Initial serum albumin level	0.96	[0.959–0.97]	< 2e − 16
Diabetes	1.42	[1.32–1.152]	< 2e − 16
Cancer	2.59	[2.35–2.85]	< 2e − 16
BMI	0.99	[0.979–0.992]	1.41E − 05
Start of dialysis with a central venous catheter	1.64	[1.52–1.77]	< 2e − 16

Adjusted for: age, sex, diabetes, BMI, vascular access, congestive heart failure, coronary heart disease, heart rhythm disorders, serum albumin level, start of dialysis with a central venous catheter, dialysis modality (peritoneal dialysis or hemodialysis), cancer, emergency start of dialysis, first dialysis session in an ICU, number of nephrology visits within one year before inception.

*Reference category: Exposure < 5%.

Specific causes of mortality showed a similar pattern globally. Cardiovascular mortality, related to heart failure, or to a rhythm disorder or sudden death or hyperkalemia, was highest in the exposure group ≥ 80%, followed by the exposure group ≤ 5%, then the exposure group 50–80% and finally, the exposure group 5–50% (Supplemental Figs. 3 and 4).

**Figure 3:**
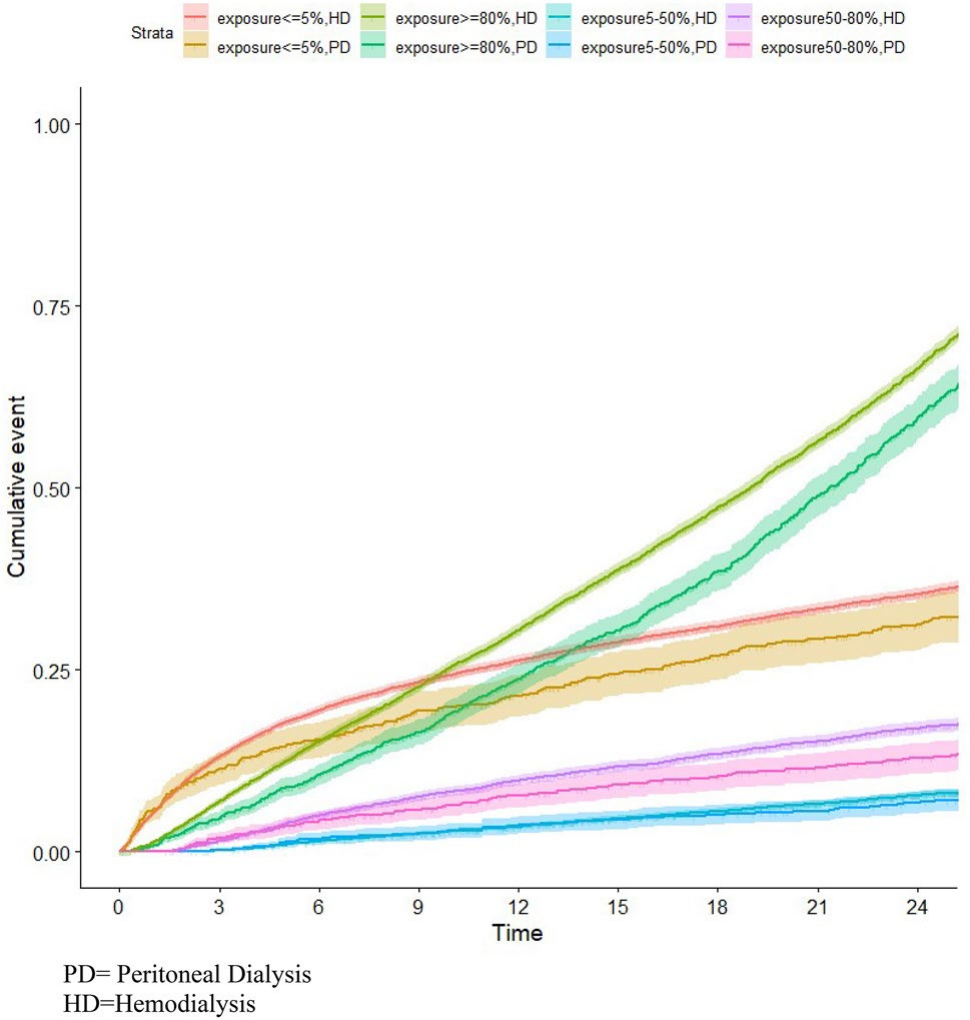
IPW-adjusted cumulative incidence (and 95% confidence interval) of death after inception according to the duration of loop diuretic exposure, stratified for dialysis modality.

**Figure 4:**
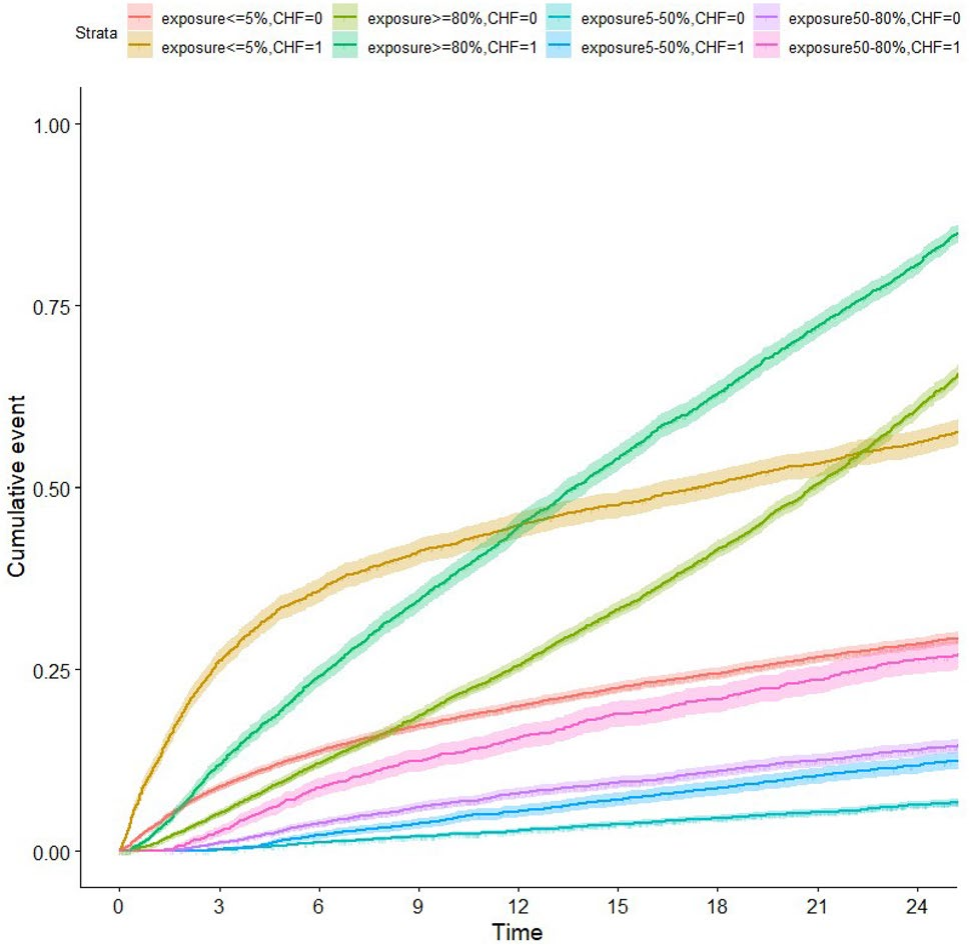
IPW-adjusted cumulative incidence (and 95% confidence interval) of death after inception, according to the duration of loop diuretic exposure, stratified for a previous history of congestive heart failure (CHF).

The IPW analysis found similar results, indicating a significant excess of mortality in the ≥ 80% exposure group. The probability of death in this group was 30.1% (95% CI 29.2–30.9) at 12 months and 66% (95% CI 64.9–67) at 24 months (Table 4).

**Table 4:** IPW-adjusted probability of death (and 95% confidence interval) at 12 months (A) and 24 months (B) after inception, according to the duration of loop diuretic exposure.

Diuretics exposure	Death probability (%)	Lower bound 0.95 CI	Upper bound 0.95 CI
(A)			
< = 5%	26	25.3	26.7
05–50%	3.5	3.2	3.8
50–80%	9.7	9	10.4
> = 80%	30.1	29.2	30.9
(B)			
< = 5%	35.2	34.4	35.9
05–50%	7.7	7.2	8.2
50–80%	16.7	15.9	17.6
> = 80%	66	64.9	67

Cumulative incidence curves stratified by dialysis modality (hemodialysis versus peritoneal dialysis) and by a history of congestive heart failure depicted similar shapes (Figs. 3 and 4). As expected, a higher mortality rate was observed in those patients with a history of heart failure in each strata of diuretics exposure. A lower incidence of mortality was found in patients on peritoneal dialysis, but this was largely explained by a higher chance for transplantation as a competing event (Supplemental Fig. 5).

### Hospitalizations

Table 5 shows hospitalization rates and length of stay in dialysis patients according to loop diuretics exposure. Patients with loop diuretics exposure ≥ 80% were significantly more often hospitalized for all causes except "hypertension" and "hyperkalemia". Hospitalizations for "CKD with dialysis" were less frequent in two groups: 5–50% exposure (1.80 [95% CI 1.76–1.84]) and 50–80% exposure (2.01 [95% CI 1.95–2.07]). There were significantly fewer stays for "pulmonary disease" in the exposure group 5–50%, and more in the exposure group ≥ 80%.

**Table 5:** Causes of hospitalizations, number and length of stay in dialysis patients according to the duration of loop diuretic exposure.

Reason for hospitalization	Exposure groups	Stays			Days		
		Number	Rate	CI 95%	Number	Rate	95% CI
Heart failure	Exposure < 5%	1483	0.60	[0.57–0.63]	17,855	7.21	[7.11–7.32]
	Exposure 05–50%	2613	0.57	[0.55–0.57]	25,051	5.46	[5.39–5.53]
	Exposure 50–80%	1685	0.75	[0.71–0.78]	16,552	7.35	[7.24–7.46]
	Exposure > 80%	2775	1.41	[1.36–1.46]	29,531	15.00	[14.83–15.17]
Acute pulmonary edema	Exposure < 5%	502	0.20	[0.19–0.22]	7489	3.03	[2.96–3.09]
	Exposure 05–50%	804	0.18	[0.16–0.19]	8668	1.89	[1.85–1.93]
	Exposure 50–80%	524	0.23	[0.21–0.25]	5893	2.62	[2.55–2.68]
	Exposure > 80%	797	0.40	[0.38–0.43]	9397	4.77	[4.68–4.87]
Pleural serositis	Exposure < 5%	124	0.05	[0.04–0.06]	1134	0.46	[0.43–0.48]
	Exposure 05–50%	171	0.04	[0.03–0.04]	1321	0.29	[0.27–0.30]
	Exposure 50–80%	93	0.04	[0.03–0.05]	805	0.36	[0.33–0.38]
	Exposure > 80%	196	0.10	[0.09–0.11]	1674	0.85	[0.81–0.89]
Hypertension	Exposure < = 5%	189	0.08	[0.07–0.09]	1658	0.67	[0.64–0.70]
	Exposure 05–50%	464	0.10	[0.09–0.11]	2989	0.65	[0.63–0.67]
	Exposure 50–80%	271	0.12	[0.11–0.13]	1905	0.85	[0.81–0.88]
	Exposure > 80%	295	0.15	[0.13–0.17]	2651	1.35	[1.30–1.40]
Myocardial infarction	Exposure < = 5%	143	0.06	[0.05–0.07]	1623	0.66	[0.62–0.69]
	Exposure 05–50%	322	0.07	[0.06–0.08]	2758	0.60	[0.58–0.62]
	Exposure 50–80%	198	0.09	[0.08–0.10]	1675	0.74	[0.71–0.78]
	Exposure > = 80%	300	0.15	[0.14–0.17]	2944	1.50	[1.44–1.55]
Pneumopathy	Exposure < 5%	780	0.32	[0.29–0.34]	8825	3.57	[3.49–3.64]
	Exposure 05–50%	1128	0.25	[0.23–0.26]	10,697	2.33	[2.29–2.38]
	Exposure 50–80%	757	0.34	[0.31–0.36]	7168	3.18	[3.11–3.26]
	Exposure > 80%	1063	0.54	[0.51–0.57]	10,827	5.50	[5.40–5.60]
Hyperkalemia	Exposure < 5%	0	0.00	0,00	0,00	0,00	0,00
	Exposure 05–50%	2	4.36E-04	[− 1.68E−04–1.04E−03]	3	0.00	[−8.61E–−04–1.39E−03]
	Exposure 50–80%	1	4.44E-04	[− 4.26E−04–1.31E−03]	7	0.00	[8.06E−04–0.01]
	Exposure > 80%	0	0.00	0.00	0.00	0.00	0.00
CKD with dialysis	Exposure < 5%	5574	2.25	[2.19–2.31]	73,598	29.74	[29.52–29.95]
	Exposure 05–50%	8249	1.80	[1.76–1.84]	80,917	17.64	[17.51–17.76]
	Exposure 50–80%	4524	2.01	[1.95–2.07]	46,034	20.44	[20.26–20.63]
	Exposure > 80%	6245	3.17	[3.09–3.25]	68,326	34.70	[34.44–34.96]
CKD without dialysis	Exposure < 5%	2574	1.04	[1.00–1.08]	22,526	9.10	[8.98–9.22]
	Exposure 05–50%	3783	0.82	[0.80–0.85]	33,539	7.31	[7.23–7.39]
	Exposure 50–80%	2047	0.91	[0.87–0.95]	15,848	7.04	[6.93–7.15]
	Exposure > 80%	3110	1.58	[1.52–1.64]	25,472	12.94	[12.78–13.10]

Rate of stays and days of stays are given per 100 patients-months.

Group with exposure < 5%: total number of months at risk = 247 512 patients-months.

Group with exposure 05–50%: total number of months at risk = 458 822 patients-months.

Group with exposure 50–80%: total number of months at risk = 225 181 patients-months.

Group with exposure > 80%: total number of months at risk = 196 879 patients-months.

In a subgroup analysis of hospital stays according to dialysis modality, HD or PD (Supplemental Tables 3 and 4), an exposure ≥ 80% was significantly associated with more stays for "heart failure", "acute pulmonary edema", and "myocardial infarction", but also other "causes unrelated to dialysis", irrespective of HD or PD. In patients on hemodialysis, the ≥ 80% exposure group also has more stays for "pulmonary disease".

## Discussion

We evaluated the effect of loop diuretics exposure on survival, specific causes of death, and hospitalizations over the two years following inception, by cross-referencing data from the nationwide REIN registry with the SNDS administrative database.

Overall, diuretic exposure was associated with a significantly lower mortality rate at two years. However, the duration of loop diuretic exposure significantly impacted outcomes among dialysis patients. Those with prolonged exposure (> 80%) experienced a markedly higher risk of death from all causes at both one and two years after inception. This effect was linear over time and quantitatively significant, with a mortality rate almost three times higher than that of the average dialysis patient. Importantly, these results were consistent across both competing risk models and IPW propensity score analyses. Additionally, prolonged exposure to loop diuretics was linked to more frequent hospitalizations for heart failure and other causes, an effect observed in both hemodialysis and peritoneal dialysis patients.

In the DOPPS study, Bragg-Gresham et al. examined the association of self-reported diuretic use with clinical outcomes among 16,420 patients receiving hemodialysis across three continents^6^. Diuretic use was associated with lower odds of interdialytic weight gain and hyperkalemia, higher odds of preserving RKF at one year, and lower cardiac mortality, with a trend toward lower all-cause mortality. The use of diuretics and the presence of RKF correlated with 32.7% of patients with RKF using diuretics versus 18.8% of patients without RKF. In a stratified analysis, an association of diuretics use with all-cause mortality was similar among patients with and without RKF, whereas diuretics use was associated with lower cardiac mortality among patients without RKF. These findings are somewhat surprising since another study in anuric HD patients showed that either low or high doses of furosemide had no significant effects on central cardiac hemodynamics^11^. Moreover, these results may be confounded by indication bias, as patients receiving diuretics were more likely to have residual kidney function^12^.

In a cohort of 11,297 patients starting hemodialysis in a large US dialysis organization, Sibbel et al. evaluated the impact of continuing versus stopping diuretics on survival, hospitalizations and intradialytic hypotension during the first year of dialysis. These authors found that continued diuretic therapy resulted in a significant reduction in the number of hospitalizations and intra-dialytic hypotensions, but not deaths at 1 year^7^. As a caveat in this study, patients with RKF were more likely to be prescribed diuretics. To address confounding by indication, the authors repeated their analyses among the subset of individuals with RKF only and found similar associations to those in the primary analysis. Even with these analyses, residual confounding was possible because the users of loop diuretics differed from non-users. Furthermore, diuretics users were more likely to receive nephrology care before dialysis initiation, which may have led to differences in decisions around dialysis initiation, peridialytic management, and long-term outcomes^13^.

Our study found a lower mortality rate at 2 years associated with loop diuretic exposure, consistent with previous findings. However, the relationship between the pattern of exposure and outcomes is particularly intriguing. We observed that patients with prolonged diuretic exposure had significantly higher death rates, increased numbers of hospital stays, and longer cumulative hospitalization times compared to those with lesser exposure. This discrepancy may stem from indication bias; we hypothesize that loop diuretics were continued in patients with persistent fluid overload or in centers aiming to preserve residual kidney function despite chronic volume expansion. Supporting this hypothesis, patients with ≥ 80% exposure had a greater history of coronary artery disease, heart failure, and rhythm disorders. This could also explain the high proportion of deaths from cardiovascular and fluid overload-related causes (e.g., pleural effusion, pulmonary disease) in this group. Importantly, the excess mortality was not limited to patients with cardiac failure and was observed regardless of a history of congestive heart failure. Alternatively, high doses of diuretics might have been maintained in patients with unstable peridialytic hemodynamics, where high ultrafiltration rates were not feasible.

Finally, we found similar results according to diuretic exposure in both hemodialysis and peritoneal dialysis patients. Within each group of diuretics exposure, mortality rates were slightly lower in peritoneal dialysis patients, which can be explained by competing risks and a higher likelihood of transplantation in these patients.

### Limitations and strengths of the study

Our study has several limitations that should be considered. As an observational study, inferring causal relationships is challenging. There may have been biases in the indications for loop diuretics that could account for some paradoxical results. Specifically, it may be difficult to distinguish between diuretic indications for fluid overload versus heart failure with preserved ejection fraction. However, our stratified analysis according to a history of heart disease showed similar outcomes.

We used an "iterative deterministic" method for matching, which allows for a strong association between two databases without directly identifying patients. The validity of this matching method has been demonstrated specifically for these two databases^8^. Some administrative hospitalization coding may be subject to questioning. For example, in our study, some dialysis patients had hospitalizations coded simply as “CKD without dialysis,” making it impossible to determine the actual cause of hospitalization.

Additionally, our study did not consider individual diuretic doses, although nearly 80% of the patients on diuretics were prescribed the 500 mg dosage of furosemide. We also did not account for associated therapies such as ACE inhibitors or beta-blockers, which could have better identified the population of patients with heart failure. However, these drugs are also widely used as antihypertensive medications, and it is uncertain whether the distinction between these groups could have been made on pharmacological grounds.

Finally, we had no information on residual diuresis and interdialytic weight gain. However, we can reasonably assume that patients with > 80% exposure to loop diuretics had preserved residual diuresis over the two-year follow-up period, while those with < 5% exposure rapidly became anuric. Similarly, patients with 5–50% and 50–80% exposure likely became progressively anuric during the follow-up period. Thus, we propose that diuretic prescription served as a proxy for residual diuresis in our cohort.

Despite these limitations, our study has several strengths. Firstly, it was based on a comprehensive contemporary cohort from the nationwide REIN registry, in which structured data were captured prospectively at initiation and during dialysis follow-up. We made adjustments to avoid confounding or indication bias by stratifying for a history of heart failure and dialysis modality and confirmed our results using IPW propensity score analysis and competing risk models. Finally, ours is the only large study to report on diuretic use and its effects in peritoneal dialysis patients.

## Conclusion

Loop diuretic exposure was associated with a lower two-year mortality rate compared to no exposure in incident dialysis patients. However, the duration of diuretic exposure had varied effects on outcomes. The longest exposure duration (> 80%) was linked to an increased risk of all-cause death at one and two years after inception and more hospitalizations for congestive heart failure or other causes. Although these results may suggest a strong indication bias in this population, they were consistent in patients with and without a history of congestive heart failure, as well as in both peritoneal dialysis and hemodialysis patients.

We propose that prolonged diuretic exposure serves as a proxy for residual diuresis at the expense of chronic fluid overload, explaining the adverse outcomes observed in this subgroup of patients. Despite the observational nature of our study, our findings underscore the need for a formal trial to evaluate the role of diuretics in managing dialysis patients.

### Ethical statement

All experiments were performed in accordance with relevant guidelines and regulations. The study was nested within the REIN registry, which has been approved by the Comité Consultatif sur le Traitement de l’Information en matière de Recherche dans le domaine de la Santé (CCTIRS), Commission National Informatique et Liberté (CNIL), and the Scientific Council of the Agence of Biomedicine.

## Supplementary Information

Supplementary Information.

## Data Availability

The data underlying this article are not publicly available but will be shared on reasonable request to the Scientific Council of the Agence of Biomedicine (Dr Cécile Couchoud, cecile.couchoud@biomedecine.fr).
