# Supplementary material for: Diuretics and mortality reduction in incident dialysis patients: a two-year observational study
Source: Sci Rep. 2024 Nov 10;14:27447. doi: 10.1038/s41598-024-65643-8 (PMC11551180; doi:10.1038/s41598-024-65643-8)
Supplement: Supplementary file 1 — Supplementary Information. [file 41598_2024_65643_MOESM1_ESM.pdf]

**Article: Diuretics and Mortality Reduction in Incident Dialysis Patients: A Two-Year Observational Study, by M. Ingwiller et al.**

**List of Supplemental Tables and Figures:**


**Supplemental Figure 1. STROBE flowchart**

**Supplemental Figure 2. Sankey diagram of the use of diuretic overtime**

**Supplemental Table 1. Characteristics of hemodialysis patients according to duration of loop diuretic exposure.**

**Supplemental Table 2. Characteristics of peritoneal patients according to duration of loop diuretic exposure.**

**Supplemental Figure 3. Cumulative incidence of death related to congestive heart failure, according to duration of loop diuretic exposure.**

**Supplemental Figure 4. Cumulative incidence of death due to hyperkalemia, heart rhytm disorders or sudden death, according to duration of loop diuretic exposure.**

**Supplemental Figure 5. Cumulative incidence function for death and transplantation as a competing risk, according to dialysis modality.**

**Supplemental Table 3. Hospitalization number, rates and lenght of stay in hemodialysis patients according to duration of loop diuretic exposure.**

**Supplemental Table 4. Hospitalization number, rates and lenght of stay in peritoneal dialysis patients according to duration of loop diuretic exposure.**

**Supplemental Figure 1. STROBE flowchart.**

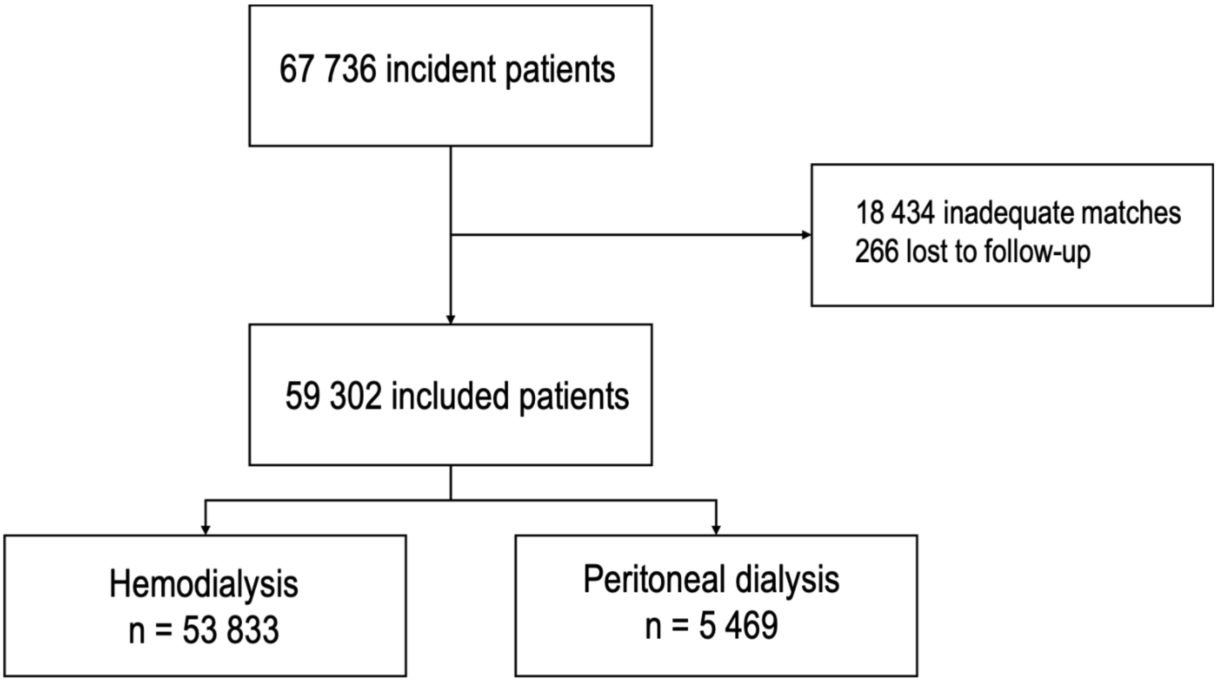

**Supplemental Figure 2. Sankey diagram of the exposure to loop diuretics over the follow-up period.**

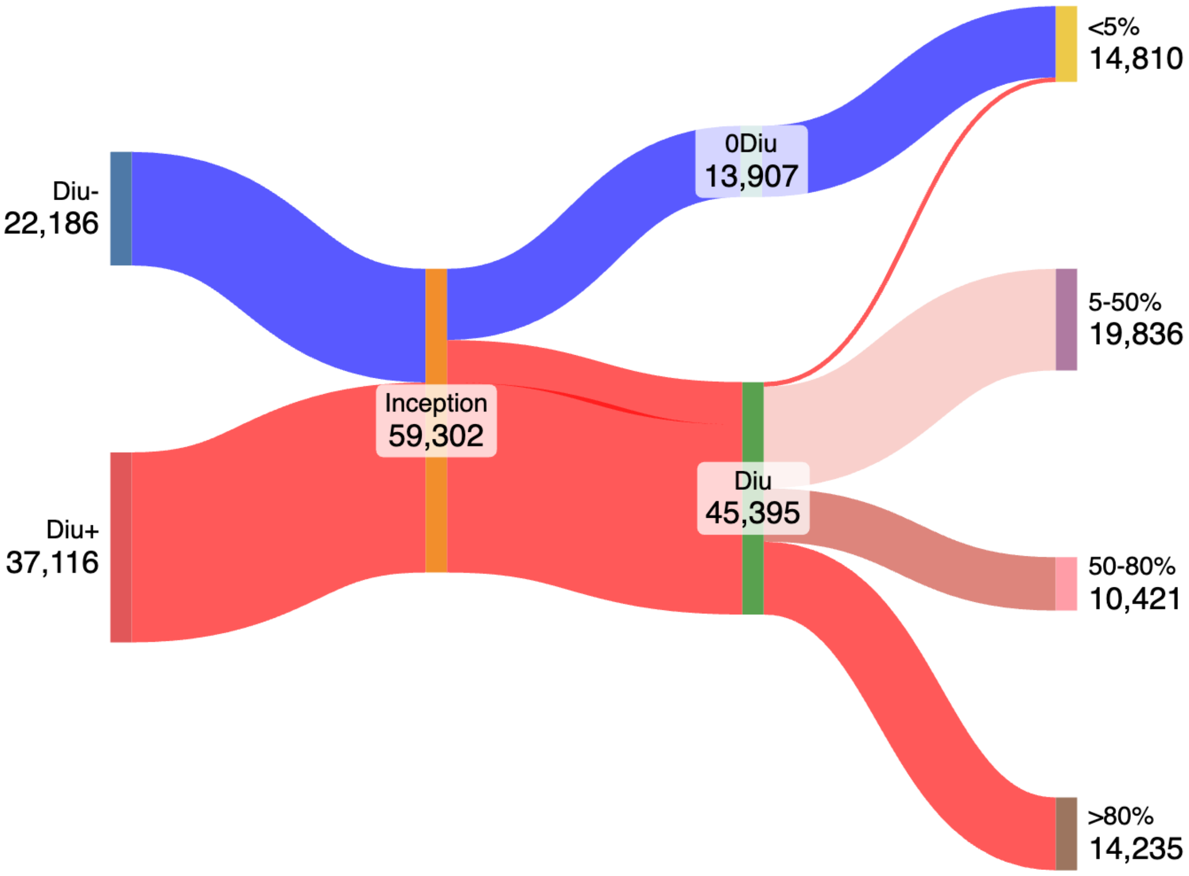


Legends: Diu+/Diu- Diuretic exposure or not at inception ; 0Diu: no diuretic exposure during the follow-up, Diu: various degrees of diuretic exposure ; <5%, 5-50%, 50-80%, >80% categories of diuretic exposure duration.

**Supplemental Table 1. Characteristics of hemodialysis patients according to the duration of loop diuretic exposure.**

| | Total | Exposure ≤ 5% | Exposure 5-50% | Exposure 50-80% | Exposure ≥ 80% |
|---|---|---|---|---|---|
| | **53833** | **13947** | **18398** | **9255** | **12233** |
| **Men** | 34350 (63.8%) | 8661 (62.1%) | 11504 (62.5%) | 6032 (65.2%) | 8153 (66.6%) |
| **Mean age** | 68.1 (±15.1) | 67.3 (±16.1) | 68.1 (±13.9) | 68.9 (±14.6) | 68.6 (±16.1) |
| **Causes of ESKD** | | | | | |
| Hypertensive nephropathy | 13940 (25.9%) | 3196 (22.9%) | 5005 (27.2%) | 2542 (27.5%) | 3197 (26.1%) |
| Diabetic nephropathy | 12479 (23.2%) | 2273 (16.3%) | 5119 (27.8%) | 2438 (26.3%) | 2649 (21.7%) |
| Glomerulonephritis | 5849 (10.9%) | 1436 (10.3%) | 1952 (10.6%) | 993 (10.7%) | 1468 (12.0%) |
| Polycystic kidney disease | 3141 (5.8%) | 999 (7.2%) | 872 (4.7%) | 503 (5.4%) | 767 (6.3%) |
| Pyelonephritis | 2210 (4.1%) | 805 (5.8%) | 697 (3.8%) | 274 (3.0%) | 434 (3.5%) |
| Other | 8631 (16.0%) | 3086 (22.2%) | 2441 (13.3%) | 1203 (13.0%) | 1901 (15.6%) |
| Unknown | 7583 (14.1%) | 2152 (15.4%) | 2312 (12.6%) | 1302 (14.1%) | 1817 (14.9%) |
| **Cardiovascular risk factors** | | | | | |
| Active smoking | 5439 (12.1%) | 1397 (12.1%) | 1908 (12.2%) | 968 (12.4%) | 1166 (11.5%) |
| Diabetes | 22882 (42.8%) | 4573 (33.1%) | 8740 (47.7%) | 4437 (48.2%) | 5132 (42.4%) |
| BMI (Kg/m²) | 26.5 (±5.-.9) | 25.1 (±5.5) | 27.5 (±6.2) | 27.0 (±5.9) | 26.2 (±5.5) |
| **Stroke / TIA** | 5724 (11.0%) | 1505 (11.2%) | 1919 (10.7%) | 994 (11.1%) | 1306 (11.1%) |
| **Coronary heart disease** | 13195 (25.4%) | 2801 (20.9%) | 4528 (25.4%) | 2456 (27.4%) | 3410 (29.0%) |
| **Heart rhythm disorders** | 11565 (22.2%) | 2644 (19.6%) | 3692 (20.6%) | 2138 (23.8%) | 3091 (26.2%) |
| **Congestive heart failure** | 13310 (25.5%) | 2936 (21.8%) | 4389 (24.5%) | 2383 (26.5%) | 3602 (30.5%) |
| **NYHA stages** | | | | | |
| Stages 1-2 | 7855 (15.4%) | 1629 (12.4%) | 2827 (16.1%) | 1432 (16.3%) | 1967 (17.1%) |
| Stages 3-4 | 4220 (8.3% | 1013 (7.7%) | 1170 (6.7%) | 746 (8.5%) | 1291 (11.2%) |
| **Peripheral arterial disease** | 12 075 (18.9%) | 2018 (14.4%) | 3333 (19.2%) | 1828 (21.0%) | 2381 (20.9%) |
| **Respiratory insufficiency** | 7690 (14.8%) | 1662 (12.4%) | 2640 (14.8%) | 1448 (16.3%) | 1940 (16.5%) |
| **Cancer** | 1654 (3.1%) | 2107 (15.6%) | 1426 (12.1%) | 1556 (8.7%) | 909 (10.1%) |
| **Behavioral disorders** | 1452 (2.9%) | 499 (3.9%) | 424 (2.5%) | 198 (2.3%) | 331 (2.9%) |
| **Walking autonomy** | | | | | |
| Total inability | 2429 (5.0%) | 1019 (8.2%) | 466 (2.8%) | 311 (3.7%) | 633 (5.8%) |
| Assistance from a 3rd party | 6186 (12.8%) | 1888 (15.2%) | 1678 (10.0%) | 1020 (12.3%) | 1600 (14.7%) |
| Autonomous walking | 39733 (82.2%) | 9527 (76.6%) | 14611 (87.2%) | 6967 (84.0%) | 8628 (79.4%) |

Legends: ESKD end-stage kidney disease ; BMI body mass index ; TIA transient ischemic attack ; NYHA New York Heart Association

**Supplemental Table 2. Characteristics of peritoneal patients according to the duration of loop diuretic exposure.**

| | Total | Exposure ≤ 5% | Exposure 5-50% | Exposure 50-80% | Exposure ≥ 80% |
|---|---|---|---|---|---|
| | **5469** | **863** | **1438** | **1166** | **2002** |
| **Men** | 3254 (59.5%) | 490 (56.8%) | 817 (56.8%) | 701 (60.1%) | 1246 (62.2%) |
| **Mean age** | 66.2 (±15.5) | 61.9 (±19.1) | 66.9 (±15.4) | 67.6 (±17.4) | 66.7 (±18.1) |
| **Causes of ESKD** | | | | | |
| Hypertensive nephropathy | 1379 (25.2%) | 180 (20.9%) | 391 (27.2%) | 305 (26.2%) | 503 (25.1%) |
| Diabetic nephropathy | 988 (18.1%) | 99 (11.5%) | 309 (21.5%) | 239 (20.5%) | 341 (17.0%) |
| Glomerulonephritis | 852 (15.6%) | 136 (15.8%) | 206 (14.3%) | 175 (15.0%) | 335 (16.7%) |
| Polycystic kidney disease | 414 (7.6%) | 101 (11.7%) | 77 (5.4%) | 64 (5.5%) | 172 (8.6%) |
| Pyelonephritis | 195 (3.6%) | 46 (5.3%) | 46 (3.2%) | 34 (2.9%) | 69 (3.4%) |
| Other | 727 (13.3%) | 142 (16.4%) | 183 (12.8%) | 140 (12.0%) | 262 (13.0%) |
| Unknown | 914 (16.7%) | 159 (18.4%) | 226 (15.7%) | 209 (17.9%) | 320 (16.0%) |
| **Cardiovascular risk factors** | | | | | |
| Active smoking | 517 (11.4%) | 82 (11.5%) | 161 (13.1%) | 113 (11.7%) | 161 (9.9%) |
| Diabetes | 1900 (35.0%) | 222 (26.1%) | 545 (38.1%) | 455 (39.2%) | 678 (34.2%) |
| BMI (Kg/m$^2$) | 25.8 (±4.8) | 24.3 (±4.4) | 26.3 (±5.0) | 26.0 (±4.9) | 25.8 (±4.6) |
| **Stroke / TIA** | 562 (10.5%) | 94 (11.3%) | 144 (10.2%) | 128 (11.2%) | 196 (10.0%) |
| **Coronary heart disease** | 1265 (23.7%) | 156 (18.6%) | 303 (21.5%) | 312 (27.4%) | 494 (25.4%) |
| **Heart rhythm disorders** | 1237 (23.1%) | 137 (16.4%) | 263 (18.7%) | 269 (23.6%) | 568 (29.0%) |
| **Congestive heart failure** | 1481 (27.7%) | 178 (21.3%) | 307 (21.7%) | 344 (30.1%) | 652 (33.4%) |
| **NYHA stages** | | | | | |
| Stages 1-2 | 678 (13.0%) | 73 (8.9%) | 167 (12.0%) | 173 (15.5%) | 265 (14.0%) |
| Stages 3-4 | 680 (13.0%) | 90 (11.0%) | 115 (8.3%) | 145 (13.0%) | 330 (17.4%) |
| **Peripheral arterial disease** | 847 (16.2%) | 100 (12.1%) | 282 (20.3%) | 318 (28.5%) | 595 (31.4%) |
| **Respiratory insufficiency** | 577 (10.8%) | 70 (8.4%) | 145 (10.2%) | 118 (10.4%) | 244 (12.5%) |
| **Cancer** | 338 (6.3%) | 52 (6.2%) | 96 (6.8%) | 70 (6.1%) | 120 (6.1%) |
| **Behavioral disorders** | 125 (2.5%) | 31 (3.9%) | 24 (1.8%) | 14 (1.3%) | 56 (3.0%) |
| **Walking autonomy** | | | | | |
| Total inability | 138 (2.8%) | 36 (4.7%) | 14 (1.1%) | 35 (3.3%) | 53 (2.9%) |
| Assistance from a 3rd party | 508 (10.3%) | 77 (10.1%) | 91 (7.0%) | 122 (11.4%) | 218 (12.1%) |
| Autonomous walking | 4288 (86.9%) | 653 (85.2%) | 1192 (91.9%) | 916 (85.4%) | 1527 (84.9%) |

Legends: ESKD end-stage kidney disease ; BMI body mass index ; TIA transient ischemic attack ; NYHA New York Heart Association

**Supplemental Figure 3. Cumulative incidence of death related to congestive heart failure (CHF), according the duration of to loop diuretic exposure.**

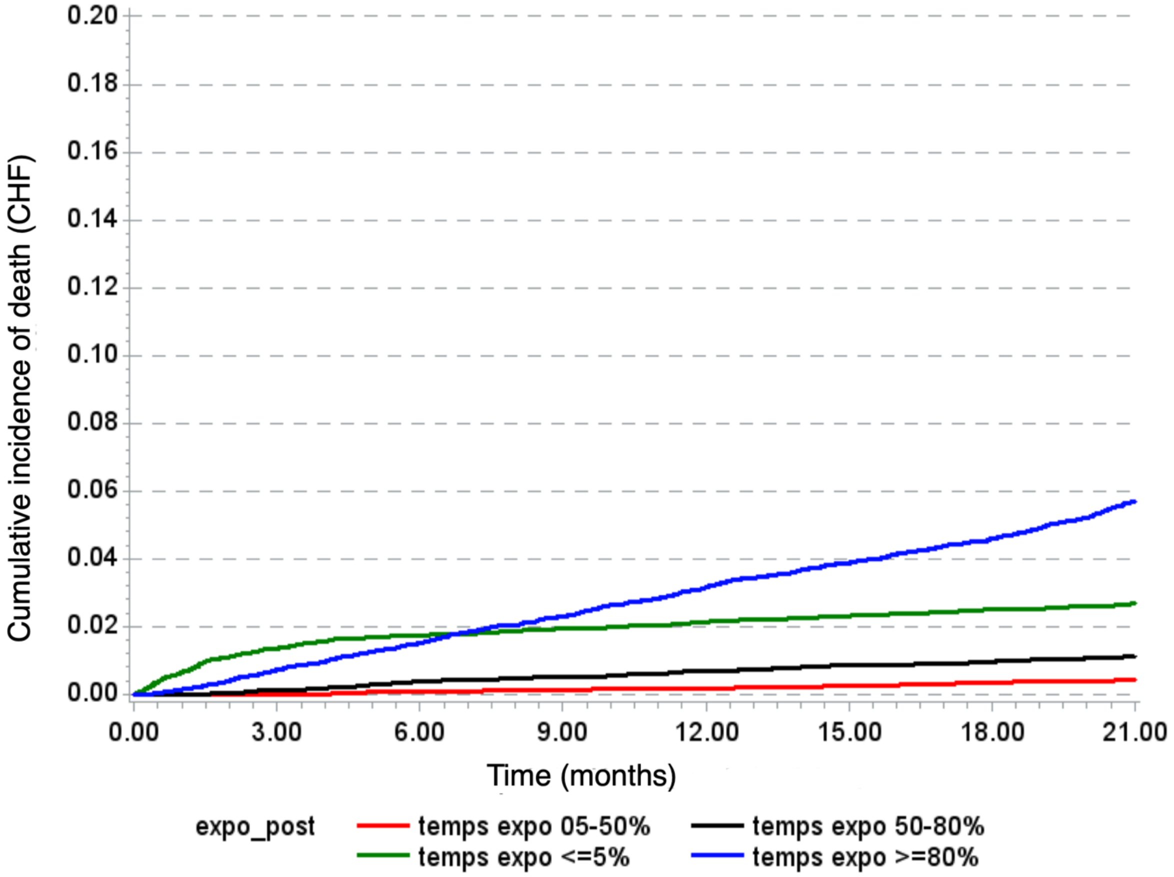

**Supplemental Figure 4. Cumulative incidence of death due to hyperkalemia, heart rhythm disorders or sudden death (SCD), according to the duration of loop diuretic exposure.**

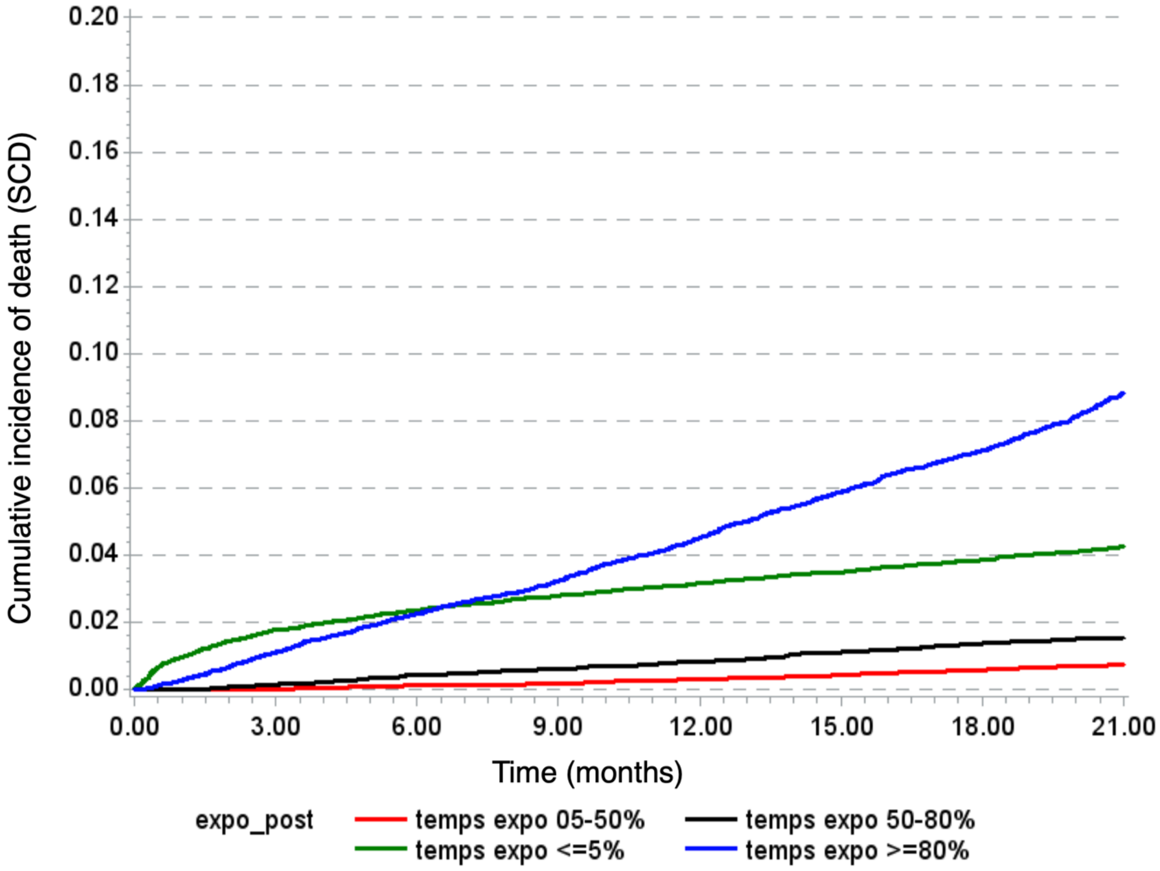

**Supplemental Figure 5. Cumulative incidence function for death and transplantation as a competing risk, according to dialysis modality.**

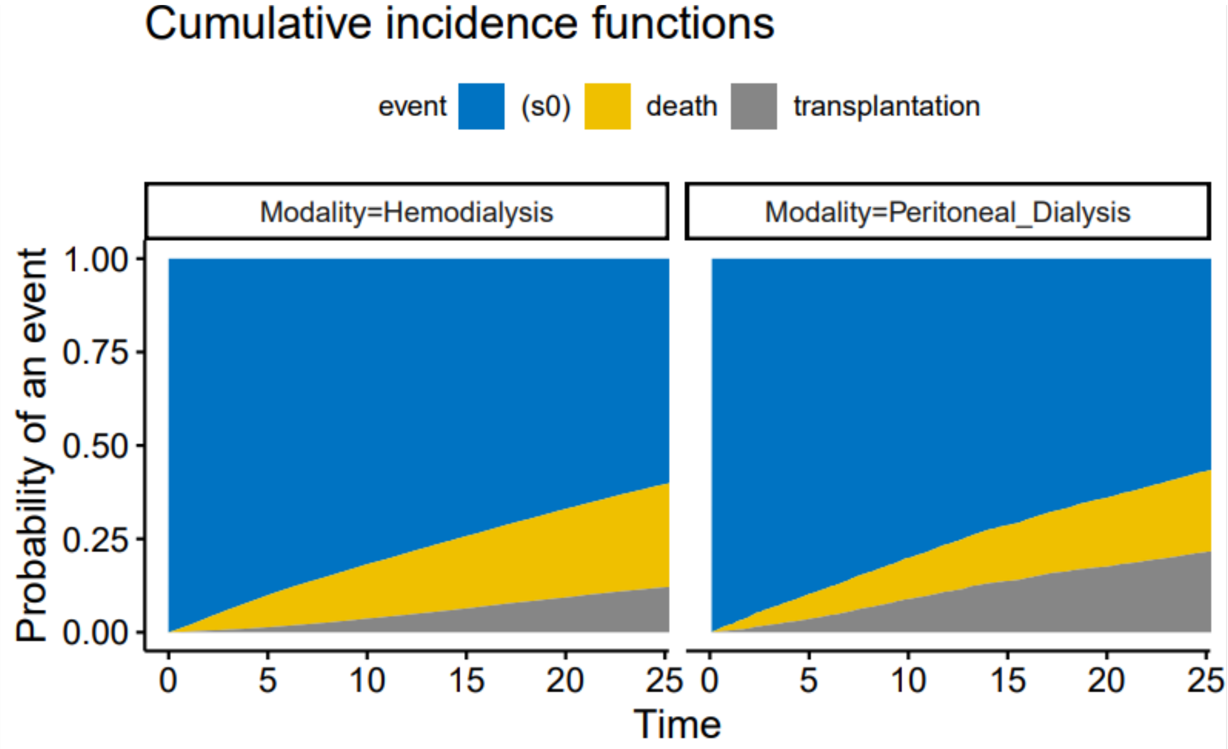

**Supplemental Table 3. Hospitalization number, rates and lenght of stay in hemodialysis patients according to the duration of loop diuretic exposure.**

| Reason for hospitalization | Time exposure | Stays | | | | Days | | |
|---|---|---|---|---|---|---|---|---|
| | | Number | Rate | 95% CI | | Number | Rate | 95% CI |
| **Heart failure** | | | | | | | | |
| | <=5% | 1431 | 0.61 | [0.58 - 0.64] | | 17074 | 7.29 | [7.19 - 7.40] |
| | 05-50% | 2456 | 0.58 | [0.55 - 0.60] | | 23550 | 5.53 | [5.46 - 5.60] |
| | 50-80% | 1544 | 0.77 | [0.73 - 0.81] | | 15216 | 7.60 | [7.48 - 7.72] |
| | >=80% | 2406 | 1.43 | [1.37 - 1.49] | | 25636 | 15.22 | [15.03 - 15.41] |
| **Acute pulmonary edema** | | | | | | | | |
| | <=5% | 495 | 0.21 | [0.19 - 0.23] | | 7390 | 3.16 | [3.09 - 3.23] |
| | 05-50% | 753 | 0.18 | [0.16 - 0.19] | | 8188 | 1.92 | [1.88 - 1.96] |
| | 50-80% | 492 | 0.25 | [0.22 - 0.27] | | 5535 | 2.76 | [2.69 - 2.84] |
| | >=80% | 731 | 0.43 | [0.40 - 0.47] | | 8601 | 5.11 | [5.00 - 5.21] |
| **Pleural serositis** | | | | | | | | |
| | <=5% | 119 | 0.05 | [0.04 - 0.06] | | 1097 | 0.47 | [0.44 - 0.50] |
| | 05-50% | 136 | 0.03 | [0.03 - 0.04] | | 1057 | 0.25 | [0.23 - 0.26] |
| | 50-80% | 77 | 0.04 | [0.03 - 0.05] | | 666 | 0.33 | [0.31 - 0.36] |
| | >=80% | 158 | 0.09 | [0.08 - 0.11] | | 1406 | 0.83 | [0.79 - 0.88] |
| **Hypertension** | | | | | | | | |
| | <=5% | 182 | 0.08 | [0.07 - 0.09] | | 1508 | 0.64 | [0.61 - 0.68] |
| | 05-50% | 407 | 0.10 | [0.09 - 0.10] | | 2650 | 0.62 | [0.60 - 0.65] |
| | 50-80% | 226 | 0.11 | [0.10 - 0.13] | | 1561 | 0.78 | [0.74 - 0.82] |
| | >=80% | 251 | 0.15 | [0.13 - 0.17] | | 2206 | 1.31 | [1.26 - 1.36] |
| **Myocardial infarction** | | | | | | | | |
| | <=5% | 139 | 0.06 | [0.05 - 0.07] | | 1523 | 0.65 | [0.62 - 0.68] |
| | 05-50% | 307 | 0.07 | [0.06 - 0.08] | | 2659 | 0.62 | [0.60 - 0.65] |
| | 50-80% | 189 | 0.09 | [0.08 - 0.11] | | 1621 | 0.81 | [0.77 - 0.85] |
| | >=80% | 268 | 0.16 | [0.14 - 0.18] | | 2616 | 1.55 | [1.49 - 1.61] |
| **Pneumopathy** | | | | | | | | |
| | <5% | 761 | 0.33 | [0.30 - 0.35] | | 8609 | 3.68 | [3.60 - 3.76] |
| | 05-50% | 1077 | 0.25 | [0.24 - 0.27] | | 10234 | 2.40 | [2.36 - 2.45] |
| | 50-80% | 684 | 0.34 | [0.32 - 0.37] | | 6323 | 3.16 | [3.08 - 3.24] |
| | >80% | 963 | 0.57 | [0.54 - 0.61] | | 9735 | 5.78 | [5.67 - 5.89] |
| **Hyperkalemia** | | | | | | | | |
| | <5% | 0 | 0 | 0 | | 0 | 0 | 0 |
| | 05-50% | 2 | 0.00 | [ 0.00 - 0.00] | | 3 | 0.00 | [ 0.00 - 0.00] |
| | 50-80% | 0 | 0 | 0 | | 0 | 0 | 0 |
| | >80% | 0 | 0 | 0 | | 0 | 0 | 0 |
| **CKD with dialysis** | | | | | | | | |
| | <=5% | 5192 | 2.22 | [2.16 - 2.28] | | 70193 | 29.99 | [29.77 - 30.21] |
| | 05-50% | 7178 | 1.69 | [1.65 - 1.72] | | 73494 | 17.26 | [17.14 - 17.39] |
| | 50-80% | 3744 | 1.87 | [1.81 - 1.93] | | 39692 | 19.83 | [19.63 - 20.02] |

| | | | | | | | | |
|---|---|---|---|---|---|---|---|---|
| | >=80% | 4961 | 2.95 | [2.86 - 3.03] | | 58760 | 34.89 | [34.61 - 35.17] |
| **CKD without dialysis** | | | | | | | | |
| | <5% | 2408 | 1.03 | [0.99 - 1.07] | | 20662 | 8.83 | [8.71 - 8.95] |
| | 05-50% | 3335 | 0.78 | [0.76 - 0.81] | | 29716 | 6.98 | [6.90 - 7.06] |
| | 50-80% | 1695 | 0.85 | [0.81 - 0.89] | | 12510 | 6.25 | [6.14 - 6.36] |
| | >80% | 2579 | 1.53 | [1.47 - 1.59] | | 21627 | 12.84 | [12.67 - 13.01] |

Hospitalization rates and days of stay are expressed per 100 patients-months

Exposure group <5%: number of months at risk = 234 067 patients-months

Exposure group 5-50%: number of months at risk = 425 782 patients-months

Exposure group 50-80%: number of months at risk = 200 194 patients-months

Exposure group >80%: number of months at risk = 168 425 patients-months

**Supplemental Table 4. Hospitalization number, rates and lenght of stay in peritoneal dialysis patients according to the duration of loop diuretics exposure.**

| Reason for hospitalization | Groupe | Stays | | | | Days | | |
|---|---|---|---|---|---|---|---|---|
| | | Number | Rate | 95% CI | | Number | Rate | IC 95% CI |
| **Heart failure** | | | | | | | | |
| | <5% | 52 | 0.39 | [0.28 - 0.49] | | 781 | 5.81 | [5.40 - 6.22] |
| | 05-50% | 157 | 0.48 | [0.40 - 0.55] | | 1501 | 4.54 | [4.31 - 4.77] |
| | 50-80% | 141 | 0.56 | [0.47 - 0.66] | | 1336 | 5.35 | [5.06 - 6.63] |
| | >80% | 369 | 1.30 | [1.16 - 1.43] | | 3895 | 13.69 | [13.26 - 14.12] |
| **Acute pulmonary edema** | | | | | | | | |
| | <5% | 7 | 0.05 | [0.01 - 0.09] | | 99 | 0.74 | [0.59 - 0.88] |
| | 05-50% | 51 | 0.15 | [0.11 - 0.20] | | 480 | 1.45 | [1.32 - 1.58] |
| | 50-80% | 32 | 0.13 | [0.08 - 0.17] | | 358 | 1.43 | [1.28 - 1.58] |
| | >80% | 66 | 0.23 | [0.18 - 0.29] | | 796 | 2.80 | [2.60 - 2.99] |
| **Pleural serositis** | | | | | | | | |
| | <5% | 5 | 0.04 | [0.00 - 0.07] | | 37 | 0.28 | [0.19 - 0.36] |
| | 05-50% | 35 | 0.11 | [0.07 - 0.14] | | 264 | 0.80 | [0.70 - 0.90] |
| | 50-80% | 16 | 0.06 | [0.03 - 0.10] | | 139 | 0.56 | [0.46 - 0.65] |
| | >80% | 38 | 0.13 | [0.09 - 0.18] | | 268 | 0.94 | [0.83 - 1.05] |
| **Hypertension** | | | | | | | | |
| | <5% | 7 | 0.05 | [0.01 - 0.09] | | 150 | 1.12 | [0.94 - 1.29] |
| | 05-50% | 57 | 0.17 | [0.13 - 0.22] | | 339 | 1.03 | [0.92 - 1.14] |
| | 50-80% | 45 | 0.18 | [0.13 - 0.23] | | 344 | 1.38 | [1.23 - 1.52] |
| | >80% | 44 | 0.15 | [0.11 - 0.20] | | 445 | 1.56 | [1.42 - 1.71] |
| **Myocardial infarction** | | | | | | | | |
| | <5% | 4 | 0.03 | [0.00 - 0.06] | | 100 | 0.74 | [0.60 - 0.89] |
| | 05-50% | 15 | 0.05 | [0.02 - 0.07] | | 99 | 0.30 | [0.24 - 0.36] |
| | 50-80% | 9 | 0.04 | [0.01 - 0.06] | | 54 | 0.22 | [0.16 - 0.27] |
| | >80% | 32 | 0.11 | [0.07 - 0.15] | | 328 | 1.15 | [1.03 - 1.28] |
| **Pneumopathy** | | | | | | | | |
| | <5% | 19 | 0.14 | [0.08 - 0.20] | | 216 | 1.61 | [1.39 - 1.82] |
| | 05-50% | 51 | 0.15 | [0.11 - 0.20] | | 463 | 1.40 | [1.27 - 1.53] |
| | 50-80% | 73 | 0.29 | [0.23 - 0.36] | | 845 | 3.38 | [3.15 - 3.61] |
| | >80% | 100 | 0.35 | [0.28 - 0.42] | | 1092 | 3.84 | [3.61 - 4.07] |
| **Hyperkalemia** | | | | | | | | |
| | <5% | 0 | 0 | 0 | | 0 | 0 | 0 |
| | 05-50% | 0 | 0 | 0 | | 0 | 0 | 0 |
| | 50-80% | 2 | 1 | [0.00 - 0.00] | | 7 | 0.03 | [0.01 - 0.05] |
| | >80% | 0 | 0 | 0 | | 0 | 0 | 0 |
| **CKD with dialysis** | | | | | | | | |
| | <5% | 382 | 2.84 | [2.56 - 3.13] | | 3405 | 25.33 | [24.48 - 26.18] |
| | 05-50% | 1071 | 3.24 | [3.05 - 3.44] | | 7423 | 22.47 | [21.96 - 22.98] |
| | 50-80% | 780 | 3.12 | [2.90 - 3.34] | | 6342 | 25.38 | [24.76 - 26.01] |
| | >80% | 1284 | 4.51 | [4.27 - 4.76] | | 9566 | 33.62 | [32.95 - 34.29] |

| | | | | | | | | |
|---|---|---|---|---|---|---|---|---|
| **CKD without dialysis** | | | | | | | | |
| | <5% | 166 | 1.23 | [1.05 - 1.42] | | 1864 | 13.86 | [13.23 - 14.49] |
| | 05-50% | 448 | 1.36 | [1.23 - 1.48] | | 3823 | 11.57 | [11.20 - 11.94] |
| | 50-80% | 352 | 1.41 | [1.26 - 1.42] | | 3338 | 13.36 | [12.91 - 13.81] |
| | >80% | 531 | 1.87 | [1.71 - 2.02] | | 3845 | 13.51 | [13.09 - 13.94] |

Hospitalization rates and days of stay are expressed per 100 patients-months

Exposure group <5% : number of months at risk = 234 067 patients-months

Exposure group 5-50% : number of months at risk = 425 782 p patients-months

Exposure group 50-80% : number of months at risk = 200 194 patients-months

Exposure group >80% : number of months at risk = 168 425 patients-months
